# Regionally specific *TSC1* and *TSC2* gene expression in tuberous sclerosis complex

**DOI:** 10.1038/s41598-018-31075-4

**Published:** 2018-09-06

**Authors:** Yi Li, Matthew J. Barkovich, Celeste M. Karch, Ryan M. Nillo, Chun-Chieh Fan, Iris J. Broce, Chin Hong Tan, Daniel Cuneo, Christopher P. Hess, William P. Dillon, Orit A. Glenn, Christine M. Glastonbury, Nicholas Olney, Jennifer S. Yokoyama, Luke W. Bonham, Bruce Miller, Aimee Kao, Nicholas Schmansky, Bruce Fischl, Ole A. Andreassen, Terry Jernigan, Anders Dale, A. James Barkovich, Rahul S. Desikan, Leo P. Sugrue

**Affiliations:** 1 Neuroradiology Section, Department of Radiology and Biomedical Imaging, University of California, San Francisco, San Francisco, CA 94143 USA; 2 Department of Psychiatry, Washington University, St. Louis, MO 63110 USA; 3 Department of Cognitive Sciences, University of California, San Diego, La Jolla, CA 92093 USA; 4 Memory and Aging Center, Department of Neurology, University of California, San Francisco, San Francisco, CA 94158 USA; 5 Athinoula A. Martinos Center, Harvard Medical School, Charlestown, MA 02129 USA; 6 Health Science and Technology Program and Computer Science and Artificial Intelligence Laboratory, Massachusetts Institute of Technology, Boston, MA 02139 USA; 7 NORMENT Institute of Clinical Medicine, University of Oslo and Division of Mental Health and Addiction, Oslo University Hospital, Oslo, Norway; 8 Departments of Radiology and Neurosciences, University of California, San Diego, La Jolla, California United States of America; 9 Departments of Neurology and Pediatrics, University of California, San Francisco, San Francisco, CA 94143 USA

## Abstract

Tuberous sclerosis complex (TSC), a heritable neurodevelopmental disorder, is caused by mutations in the *TSC1* or *TSC2* genes. To date, there has been little work to elucidate regional *TSC1* and *TSC2* gene expression within the human brain, how it changes with age, and how it may influence disease. Using a publicly available microarray dataset, we found that *TSC1* and *TSC2* gene expression was highest within the adult neo-cerebellum and that this pattern of increased cerebellar expression was maintained throughout postnatal development. During mid-gestational fetal development, however, *TSC1* and *TSC2* expression was highest in the cortical plate. Using a bioinformatics approach to explore protein and genetic interactions, we confirmed extensive connections between TSC1/TSC2 and the other genes that comprise the mammalian target of rapamycin (mTOR) pathway, and show that the mTOR pathway genes with the highest connectivity are also selectively expressed within the cerebellum. Finally, compared to age-matched controls, we found increased cerebellar volumes in pediatric TSC patients without current exposure to antiepileptic drugs. Considered together, these findings suggest that the cerebellum may play a central role in TSC pathogenesis and may contribute to the cognitive impairment, including the high incidence of autism spectrum disorder, observed in the TSC population.

## Introduction

Tuberous sclerosis complex (TSC), a heritable neurodevelopmental disorder, is caused by mutations in the *TSC1* or *TSC2* genes, which encode the proteins hamartin and tuberin^1^. Together, these proteins form a complex that inhibits the growth-regulating mammalian target of rapamycin (mTOR) pathway^2^. Although the genes and molecular pathways associated with hamartin and tuberin are well known, comparatively less is known about regional patterns of *TSC1* and *TSC2* gene expression within the human brain.

Accumulating evidence suggests that patterns of regional gene expression guide human neurodevelopment^3^. Just as regional *TSC1* and *TSC2* gene expression patterns contribute to the development of tumors and hamartomas elsewhere in the body, we hypothesized that regional *TSC1/TSC2* expression in the brain may influence neuroanatomic and cognitive changes associated with tuberous sclerosis. To explore this hypothesis, in this paper we examine patterns of *TSC1* and *TSC2* expression within the normal human brain and compare them to changes in regional brain morphology in patients with TSC.

## Results

### TSC1 and TSC2 expression is selectively increased within the cerebellum and neocortex

**Figure 1:**
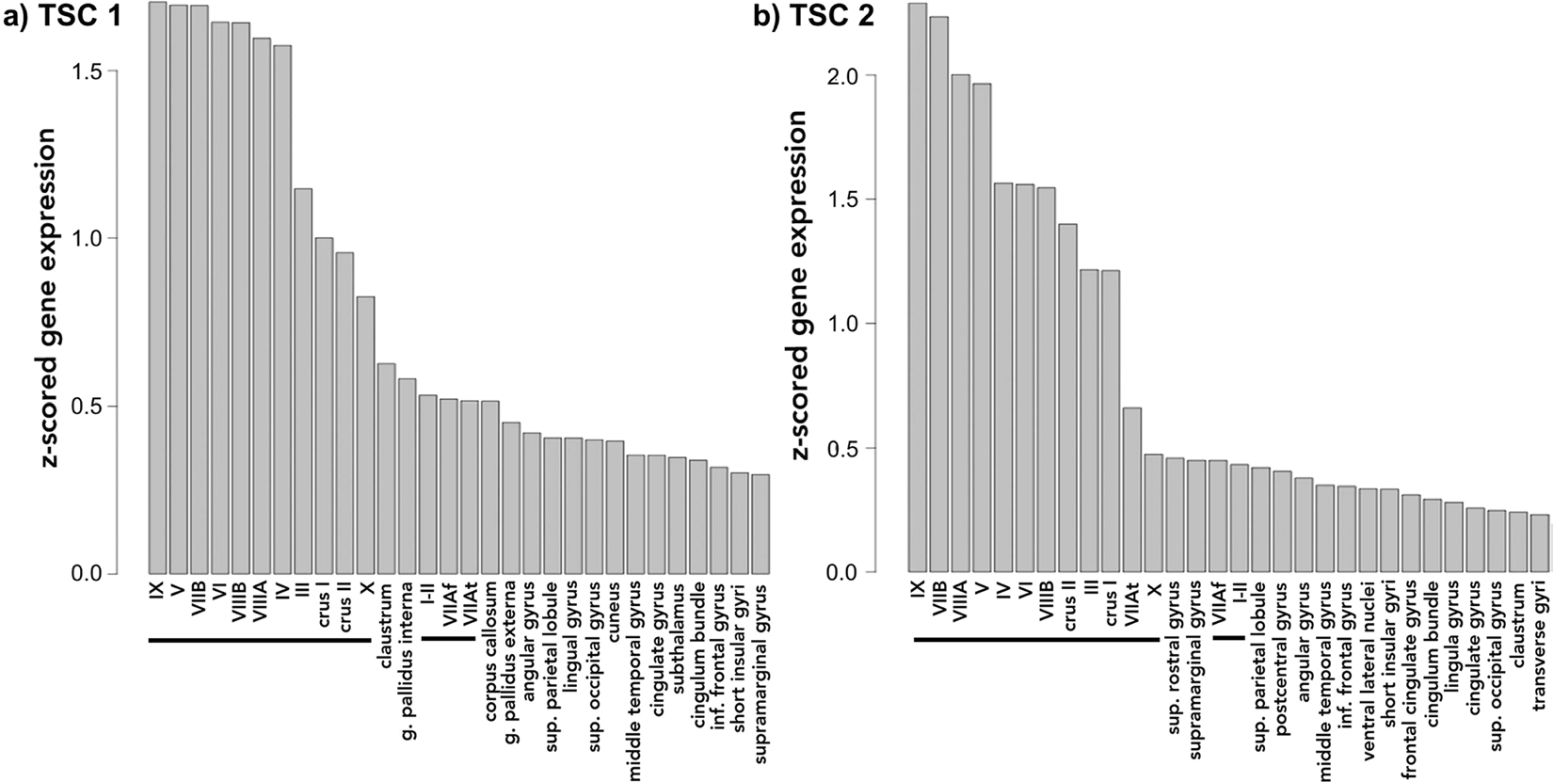
Bar plots illustrating the thirty brain regions demonstrating highest mean regional transcript levels for *TSC1* **(a)** and *TSC2* **(b)** using data from the Allen Brain Science Institute (www.brain-map.org)^29^. Values represent z-scores averaged across the individual probes and across the 6 postmortem subjects. The horizontal black line highlights cerebellar regions.

**Figure 2:**
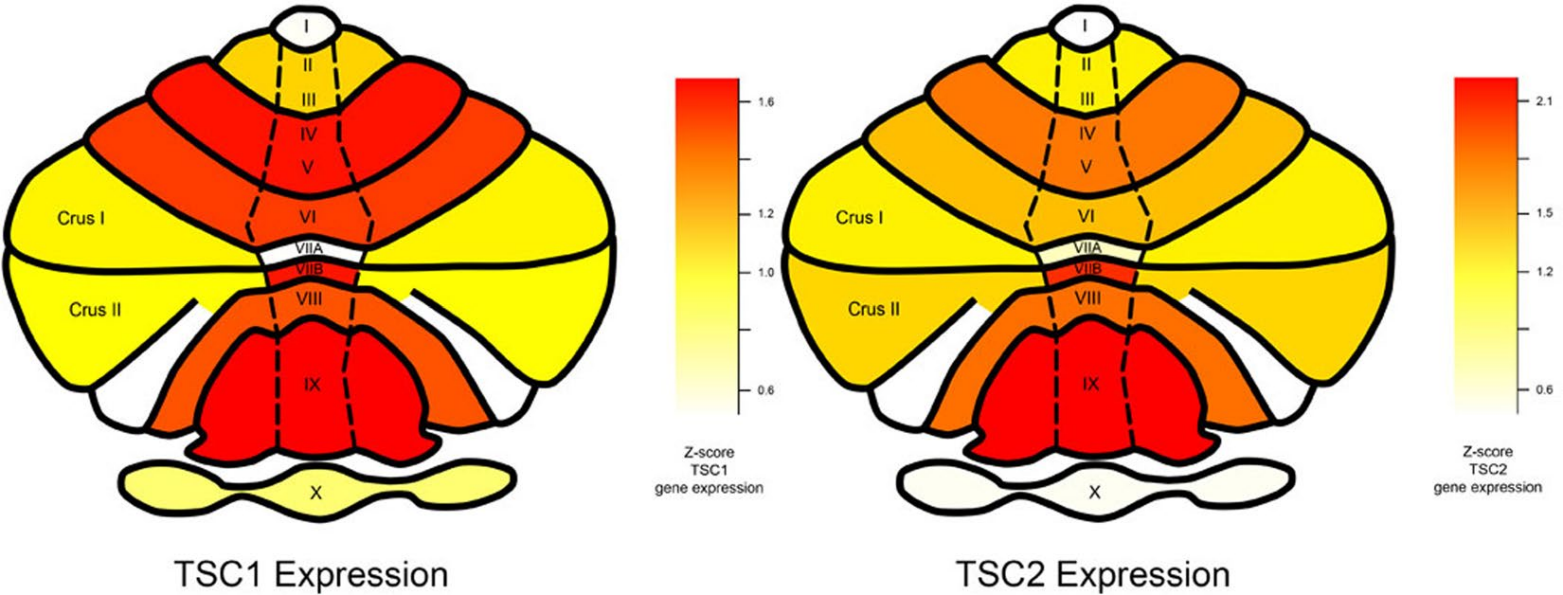
Average regional cerebellar gene expression of *TSC1* (left) and *TSC2* (right) from all 6 Allen Brain Science Institute postmortem brains (www.brain-map.org)^29^ mapped onto a pictorial heat-map representation of the cerebellar lobules and crus. As shown in the color scale, red indicates relatively higher average expression (z-scores) and yellow indicates relatively lower average expression.

**Figure 3:**
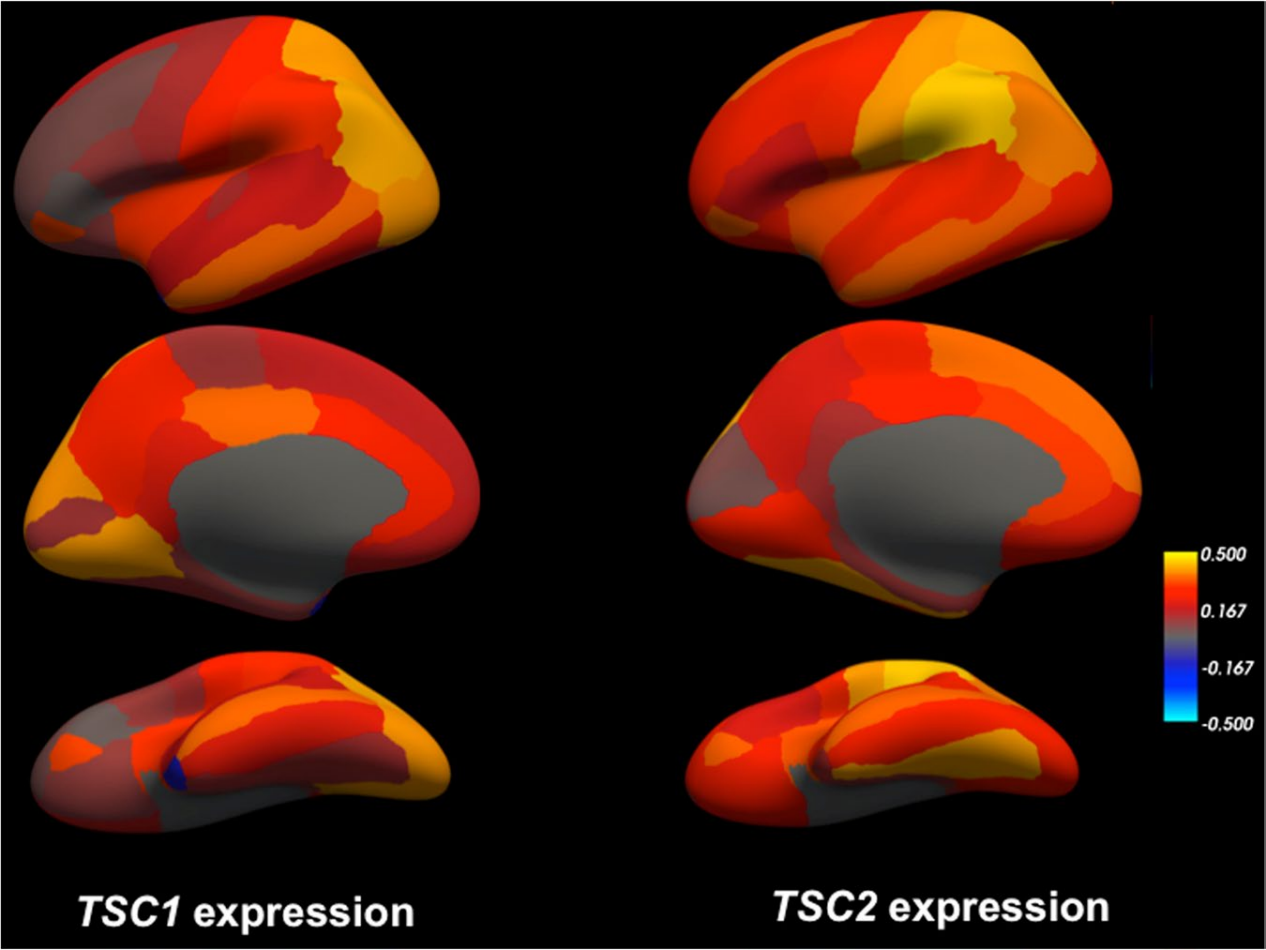
Average regional gene expression of *TSC1* (left) and *TSC2* (right) from all 6 Allen Brain Science Institute postmortem brains (www.brain-map.org)^29^ mapped into a three-dimensional reconstruction (‘inflated’ view) of the gray/white matter boundary of the cerebral cortex (fsaverage subject from FreeSurfer). Top panel illustrates the lateral view, middle panels illustrate the medial view, and bottom panels illustrate the ventral view of the left cerebral hemisphere. As shown in the color scale, yellow indicates relatively higher expression (z-scores) and blue indicates relatively lower expression.

**Figure 4:**
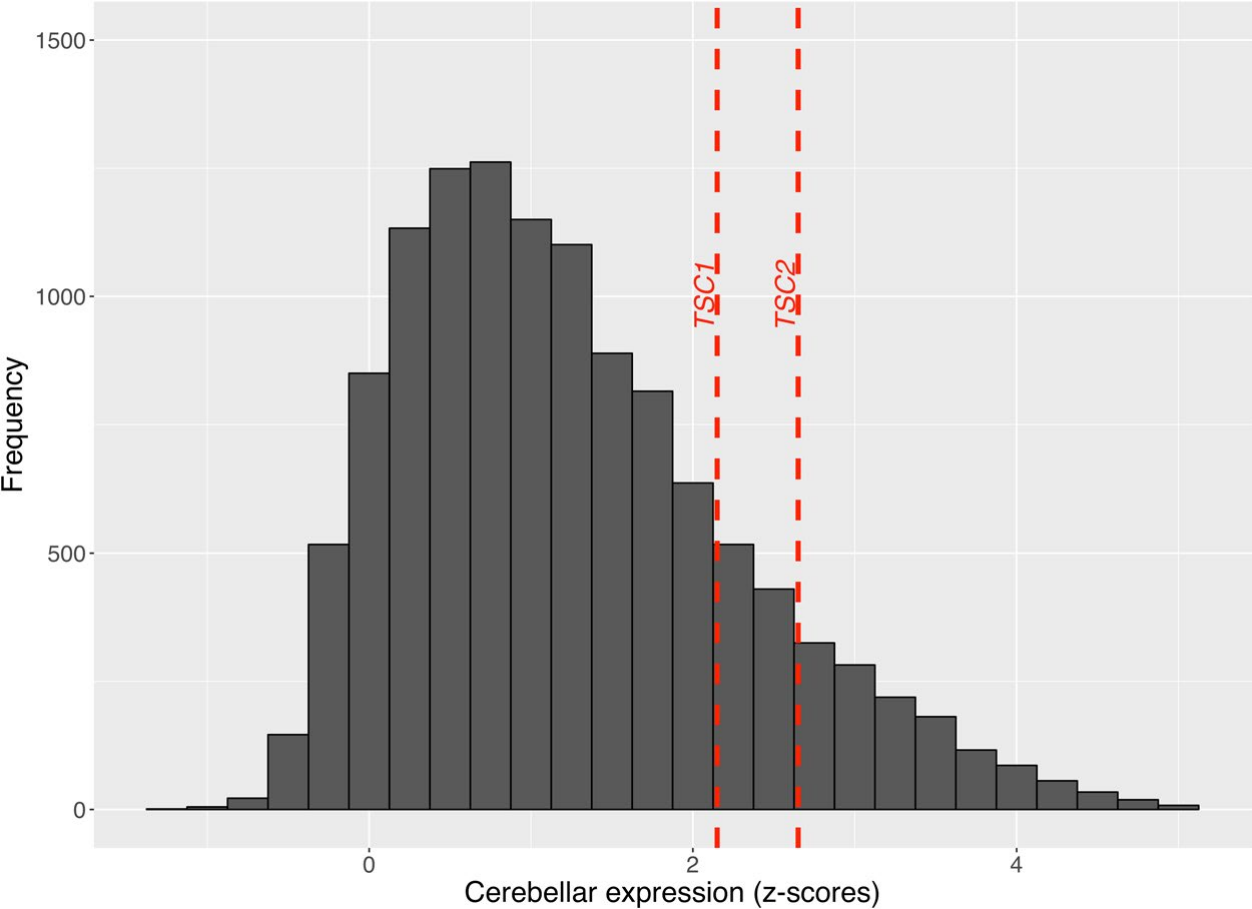
Histogram showing the relative (z-scored) cerebellar expression of all 12000 + genes from the Allen Brain Science Institute database (www.brain-map.org)^29^, depicted by frequency (y-axis) of each z-score of relative cerebellar expression (x-axis). The dashed vertical lines depict z-scores of relative cerebellar expression for TSC1 and TSC2 compared to all other genes. The rightward shift of this distribution reveals that expression of many genes is relatively increased in the cerebellum compared to other brain regions; however, the location of *TSC1* and *TSC2* within the positive tail of the distribution confirms their greater regional specificity.

Using a publicly available microarray dataset from the Allen Brain Sciences Institute (www.brain-map.org), we investigated regional gene expression of *TSC1* and *TSC2* with the goal of leveraging patterns of gene expression to better understand the TSC phenotype (see Methods). Across all brain regions, we found significantly elevated expression within all cerebellar lobules and crus I and II compared to other brain regions (*TSC1* t-test p value = 1.16 × 10^−15^ and *TSC2* t-test p value = 2.38 × 10^−20^) (Figs 1 and 2). Compared to subcortical regions (such as the hippocampus), we also found relatively elevated expression within certain cortical areas: the supramarginal gyrus, angular gyrus and superior parietal lobule (*TSC1* t-test p value = 3.30 × 10^−12^ and *TSC2* t-test p value = 1.61 × 10^−11^) (Figs 1 and 3). Finally, *TSC1* and *TSC2* expression within all cerebellar lobules and crus was significantly elevated, even when compared to those supratentorial areas with relatively high expression (*TSC1* t-test p value = 2.80 × 10^−8^ and *TSC2* t-test p value = 1.70 × 10^−12^). To evaluate whether elevated cerebellar expression is specific to *TSC1* and *TSC2*, we computed z-scores of expression levels across brain areas for each of the 12,000 + genes within the Allen Brain Atlas database and then examined the distribution of relative cerebellar expression across all genes (Fig. 4). The rightward shift of this distribution reveals that expression of many genes is relatively increased in the cerebellum compared to other brain regions; however, the location of *TSC1* and *TSC2* within the positive tail of the distribution confirms their greater regional specificity.

We assessed the reproducibility of our cerebellar *TSC1* and *TSC2* findings using two additional independent gene expression datasets from post-mortem brains: the Genotype-Tissue Expression (GTEx) project^4^ (n = 125 disease-free adult brains) and UK Brain Expression Consortium (UKBEC)^5^ (n = 134 disease-free adult brains). Within both the GTEx and UKBEC datasets, across all evaluated regions, we again found the highest *TSC1* and *TSC2* expression within the cerebellum (Supplemental Figs 1 and 2). Thus, three independent gene expression datasets confirm that that *TSC1* and *TSC2* gene expression is selectively elevated within the cerebellum.

### Assessing regional TSC1 and TSC2 expression during post-natal development

**Figure 5:**
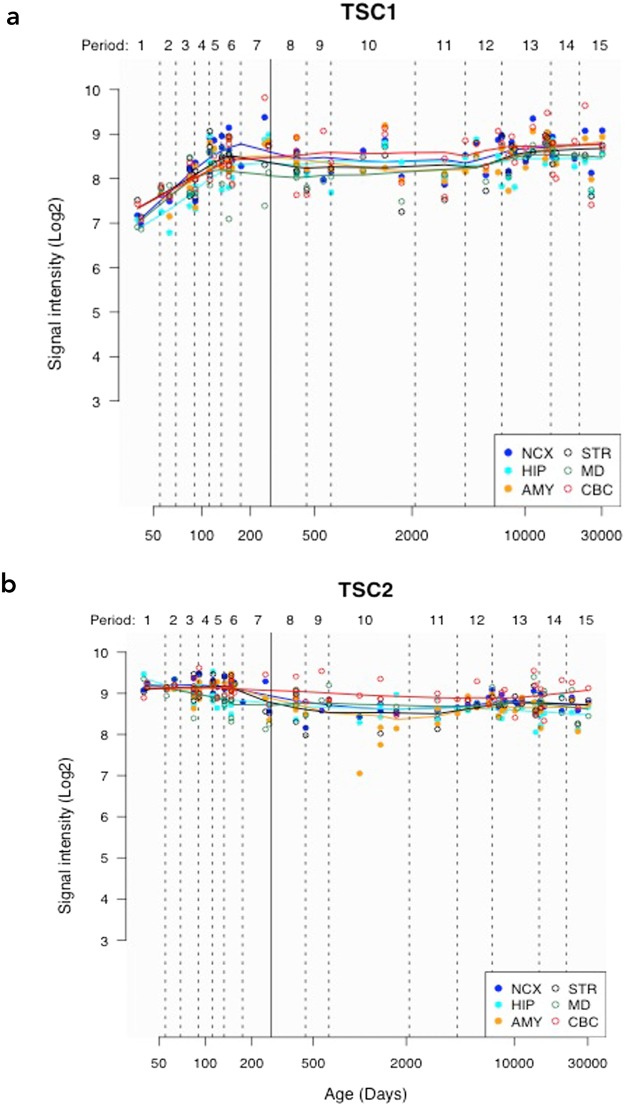
Line plots showing *TSC1* **(a**) and *TSC2* **(b)** gene expression over time, using data from the Human Brain Transcriptome (http://hbatlas.org/). NCX = neocortex, MD = medial dorsal thalamus, CBC = cerebellar cortex, HIP = hippocampus, STR = striatum and AMY = amygdala. ‘Y-axis shows relative gene expression as Log2 signal intensity assessed from hybridization of cDNA generated from extracted RNA to Affymetrix Human Exon microarrays as detailed in Kang *et al*., Nature 2011. ‘Period’ refers to periods of human development and adulthood defined in that original paper and chosen to emphasize the timing and progression of major neurodevelopmental events in the Cerebral Cortex.’ X-axis shows post-conception age in days; the solid vertical line indicates the transition between pre-natal and post-natal periods.

We additionally investigated *TSC1* and *TSC2* gene expression utilizing a microarray expression data set focused on the developing brain (Kang HJ *et al*., Nature 2011) (see Methods). Across all evaluated regions, we found the highest *TSC1* and *TSC2* expression within the cerebellar cortex (CBC) (Fig. 5). Compared to the medial dorsal thalamus, which showed the lowest expression, across all time points, we found significantly elevated expression within the CBC for *TSC1* (paired t-test p-value = 0.002) and *TSC2* (paired t-test p-value = 2.7 × 10^−5^). Using a repeated measure ANOVA, within the CBC, we found marginal evidence for differential *TSC1* expression over time (12 PCW to 83 years) (F = 4.03, p = 0.05), but no evidence for differential *TSC2* expression (F = 0.03, p = 0.58) over time.

### Selectively elevated TSC1 and TSC2 expression within the fetal cortical plate and subplate

**Figure 6:**
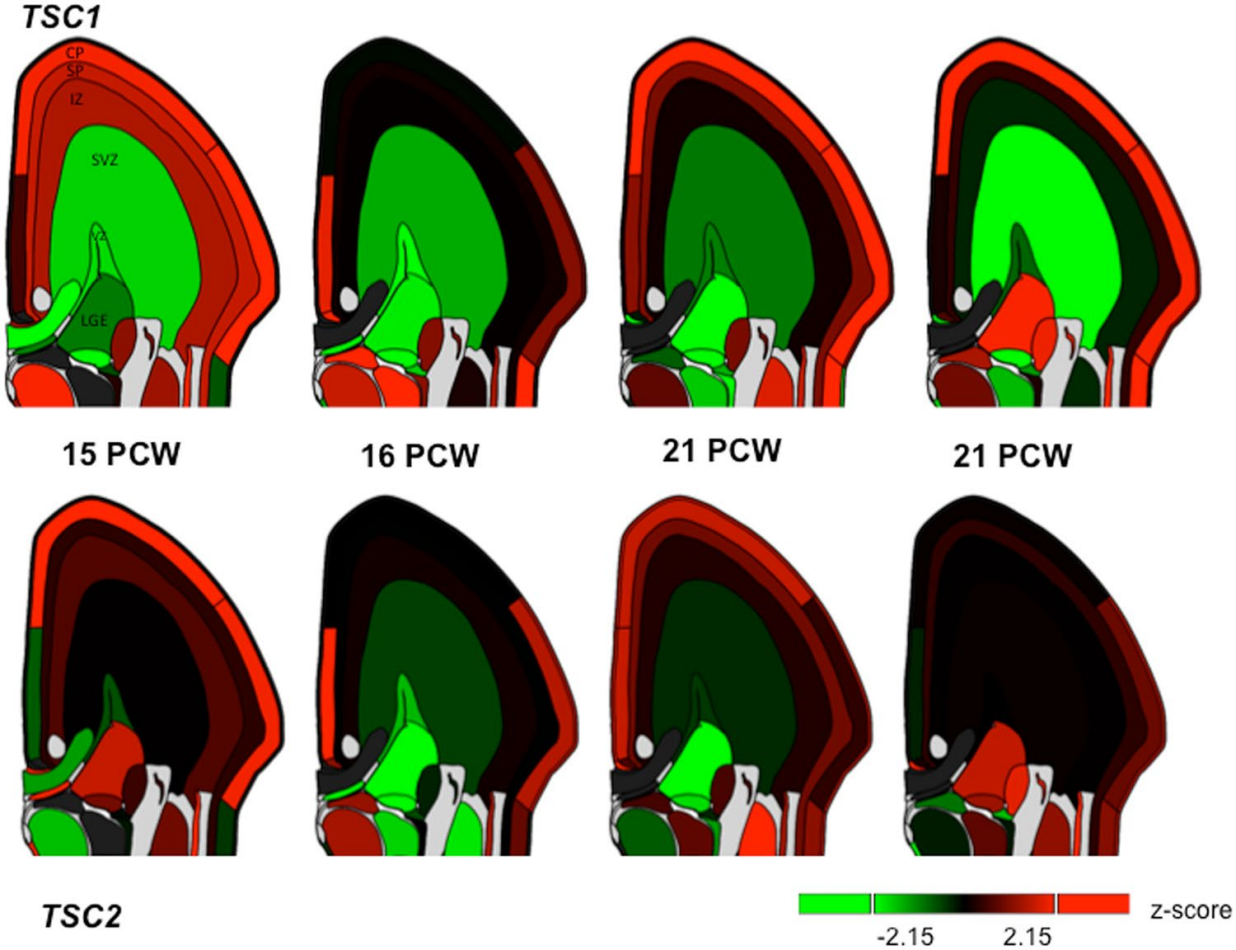
Regional *TSC1* and *TSC2* RNA expression within four fetal brains at 15, 16 and 21 weeks post-conception (PCW) from the Allen Brain Sciences Institute database (http://www.brainspan.org/lcm/)^31^. VZ = ventricular zone, SZ = subventricular zone, IZ = intermediate zone, SP = subplate, and CP = cortical plate.

The pathogenic variants and consequent alterations in molecular pathways that result in TSC are set into motion during fetal development; we therefore next examined *TSC1* and *TSC2* expression *in utero* using data from the Allen Brain Sciences Institute. Assessing mean *TSC1* and *TSC2* expression across the 4 mid-gestational fetal brains and across all fetal mitotic and post-mitotic zones, we found the highest expression within the cortical plate (CP) and lowest expression within the subventricular zone (SZ) (Fig. 6). In comparison to the SZ, the CP demonstrated significantly elevated expression (*TSC1* t-test p-value = 2.0 × 10^−30^ and *TSC2* t-test p-value = 0.002). The subplate (SP), another post-mitotic zone showing high expression, also demonstrated significantly higher expression compared to SZ (*TSC1* t-test p-value = 1.3 × 10^−8^ and *TSC2* t-test p-value = 0.003). Compared to SP, expression within CP was significantly elevated for *TSC1* (t-test p-value = 3.2 × 10^−6^) but not for *TSC2* (t-test p-value = 0.82). Thus, *TSC1* and, to a lesser extent, *TSC2* gene expression is highest within the cortical plate in the developing fetal brain.

### Protein-protein and co-expression networks for TSC1 and TSC2

To evaluate potential genetic interactions, co-expression, co-localization and protein domain similarity for *TSC1* and *TSC2*, we used GeneMANIA (www.genemania.org), a bioinformatics method for functional prediction of gene networks based on genes and gene sets^6^. In addition to visualizing the composite gene network, we also assessed the weights of individual network components^7^.

**Figure 7:**
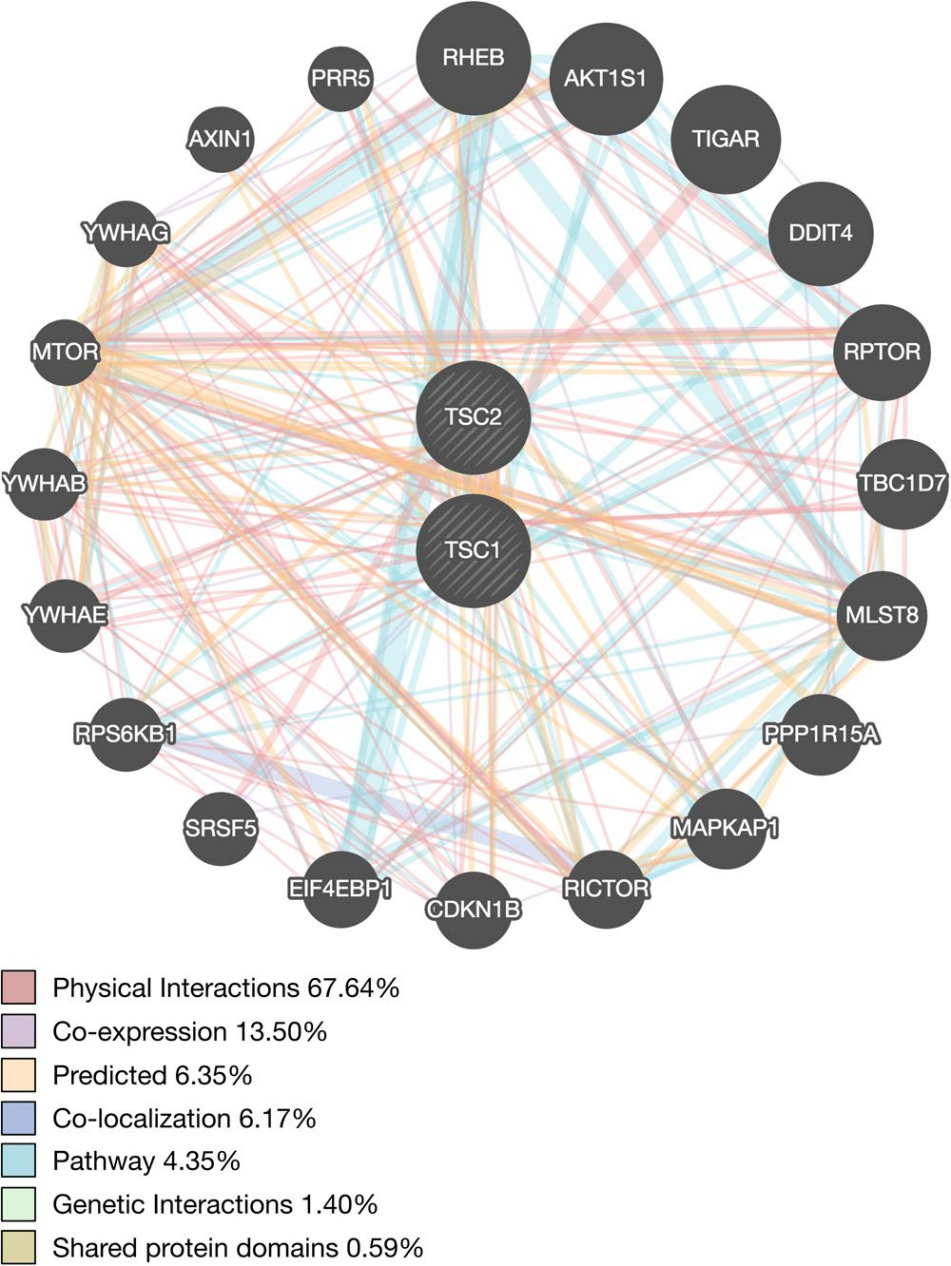
Network interaction graph illustrating physical interactions (pink), co-expression (purple), predicted (orange), pathway (aqua), co-localization (blue), genetic interactions (green) and shared protein domains (khaki) for *TSC1* and *TSC2* assessed using GeneMANIA (www.genemania.org).

We found that *TSC1* and *TSC2* showed strong physical interactions, predicted interaction, and co-expression with several genes and proteins (Fig. 7). As expected, across all analyses, we found the strongest network weights (>0.60) between *TSC1*, *TSC2* and other members of the P13K/AKT/mTOR pathway, namely *mTOR*, *c12orf5/TIGAR*, *RPTOR*, *MLST8*, *AKT1S1*, *RHEB*, *RPS6KB1*, *DEPTOR*, *EIF4EBP1* and *RICTOR* (See supplemental materials for details about these individual genes and their protein products). We subsequently evaluated the regional expression patterns of several of these mTOR pathway genes using the GTEx database^4^. We found that multiple mTOR pathway genes from the *TSC1* and *TSC2* gene network, including RICTOR, MLST8, RPS6KB1 and mTOR itself, also demonstrate selectively elevated cerebellar expression (Supplemental Fig. 3a–d), suggesting molecular enrichment for the mTOR pathway as a whole within the cerebellum.

### *In vivo* morphometric analysis of cerebellar volume in pediatric patients with TSC

To evaluate potential *in vivo* cerebellar abnormalities in pediatric patients with TSC we performed morphometric analysis of cerebellar lobule volumes in a group of 28 patients with TSC from our institution (see Methods).

**Figure 8:**
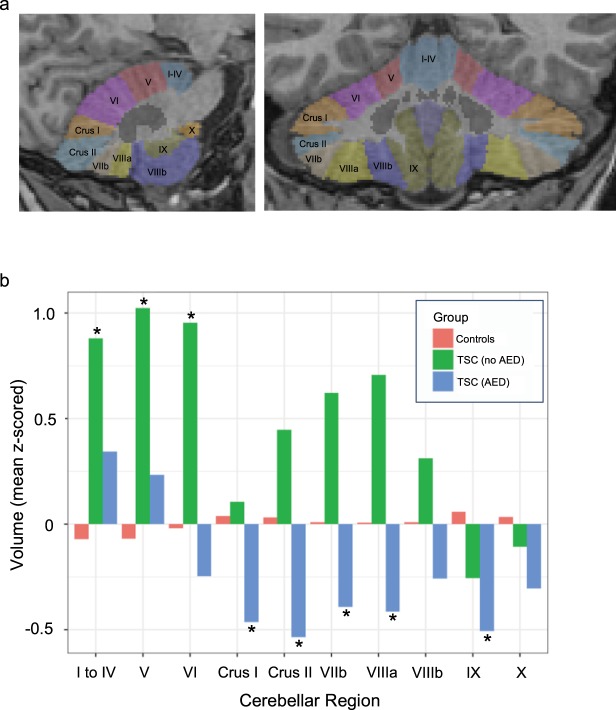
(**a**) Sagittal (left) and Coronal (right) images of a representative subject’s brain showing individual cerebellar lobules segmented using the SUIT (‘spatially unbiased infra-tentorial template‘) software package^33^. (**b**) Bar graphs show mean volumes of each cerebellar region for each of 3 groups: Controls (red) (n = 200), TSC patients not taking anti-epileptic drugs (AEDs) green (n = 10), and TSC patients taking AEDs (blue) (n = 18). Volumes of each cerebellar region were z-scored across all subjects/groups before computing group averages. Asterisks (*) highlight regions/groups where TSC volumes differ significantly from those of the control group at a nominal p-value < 0.05 derived from a linear regression model of group effects that controls for age and gender (see Supplemental Table 1a,b for individual model results).

Epilepsy is common amongst TSC patients and anti-epileptic drug (AED) exposure is known to correlate with cerebellar atrophy, either as a direct effect or as an indirect consequence of seizure severity^8,9^. For this reason, we separated our TSC population into two groups according to history of active anti-epileptic drug use at the time of MRI scanning, assessed through review of medical records. Others have previously reported cerebellar cortical volume loss in patients with TSC^10^. In our TSC population, the 18 TSC patients with exposure to AEDs had decreased cerebellar volumes compared to normal age-matched controls (Fig. 8b, blue bars), with most areas reaching significance at nominal p-value < 0.05 (derived from a linear regression model that controlled for age and gender, see Supplemental Table 1a). Interestingly, however, the 10 TSC patients without exposure to AEDs had significantly *increased* cerebellar volumes compared to controls in multiple cerebellar lobules and showed a consistent trend towards increased volumes across almost all regions (Fig. 8b, green bars; Supplemental Table 1b).

## Discussion

In this study, we found that *TSC1* and *TSC2*, the genes responsible for TSC, are expressed in the brain in a regionally specific pattern that changes throughout development. *In utero*, *TSC1*, and to a lesser extent, *TSC2* expression is highest in the cortical plate, the developmental zone which, during mid-fetal development, has enriched expression of gene networks implicated in autism spectrum disorders (ASD)^11^. During post-natal development, when symptoms of TSC tend to initially manifest, *TSC1* and *TSC2* gene expression is highest in the cerebellum, suggesting that the cerebellum may play an important role in TSC pathogenesis. Using gene expression network analysis, we confirm that *TSC1* and *TSC2* are strongly co-expressed with other genes of the P13K/AKT/mTOR pathway. Moreover, in adults we find that these other mTOR pathway genes also demonstrate elevated cerebellar expression, suggesting molecular enrichment for mTOR within the cerebellum.

Correlating the observed *TSC1* and *TSC2* gene expression data with structural changes, we found, as others have reported^10^, a decrease in size of cerebellar lobules in TSC patients compared to age matched controls. However, this effect was only present in TSC patients with current exposure to AEDs. In the absence of AED exposure, patients with TSC demonstrated larger cerebellar volumes relative to controls. These results highlight the potential confounding influence of seizure burden/AED exposure in studying the TSC population. Earlier reports of cerebellar volume loss in TSC^10^ seemed at odds with the overgrowth seen elsewhere in the body in response to mutations of the TSC complex which is an inhibitory component of the pro-growth mTOR pathway. Our results could explain this discrepancy, ultimately, however, larger longitudinal studies will be needed to better understand how regional gene expression in TSC impacts brain structure and morphology throughout development.

Prior studies have investigated the presence of cerebellar pathology in TSC and its implications for the TSC phenotype. While approximately 80% of patients with TSC have cortical tubers^12^, only 24–33% of patients have tubers in the cerebellum^13,14^. Interestingly, the presence of cerebellar tubers does not correlate with “typical” cerebellar motor symptoms, as might be expected^14^, but rather, with neurocognitive symptoms^15,16^. This finding fits with growing clinical evidence that cerebellar abnormalities can have a profound impact on cognitive development^17^, and basic research showing that the posterior lobe of the cerebellum (lobules VI, VII, VIII, IX, crus I, and crus II, collectively constituting the neo-cerebellum) is integral to cognitive and limbic regulation^18^. In normal adults, we found selective elevation of *TSC1* and *TSC2* gene expression within these same neocerebellar lobules, suggesting that regional disruption of *TSC1/TSC2*, and the mTOR pathway more generally, may contribute to the cognitive abnormalities seen in TSC.

Approximately 50% of patients with TSC fulfill criteria for autism spectrum disorder (ASD), making TSC one of the most common monogenic causes of ASD^19^. The pathogenesis of ASD in the TSC population is not well understood, however, in recent years, accumulating evidence points to the important role the cerebellum plays not only in cognition, but also in the clinical development of autism spectrum disorder (ASD). Prior neuropathological studies have suggested a loss of cerebellar Purkinje cells in individuals with ASD^20–22^. Additionally, prior imaging studies have shown abnormal cerebellar morphology and function in patients with ASD, particularly within the neo-cerebellum^23–26^. Premature infants with isolated cerebellar hemorrhage have a higher incidence of ASD compared to other premature controls^27^. Finally, mutant mice with selective deletion of *TSC1* from the cerebellum develop an autistic phenotype characterized by lack of interest in socializing, repetitive behaviors and restrictive interests^19^. These studies support our hypothesis that abnormal cerebellar development, due to a variety of causes, including abnormal regional expression of *TSC1*, *TSC2* and related gene networks, may contribute to cognitive disorders such as ASD.

In mid-gestational fetal brains, we found elevated *TSC1* and, to a lesser extent, *TSC2* expression in the cortical plate. Recent studies have implicated the mid-gestational cortical plate as a key point of convergence of multiple co-expression networks of probable ASD genes^11^. In the adult cortex we found regionally selective expression of *TSC1* and *TSC2* in the association cortex of the parietal lobe, a major area of input into the neo-cerebellum^28^. Together these cerebellar and cortical findings suggest that a cortico-cerebellar network linking association cortex to the neo-cerebellum may contribute to the pathogenesis of cognitive disorders in TSC.

Our study has limitations. The gene expression data in our paper is derived from normal adult and fetal brain tissue and not from TSC patients. Furthermore, the pediatric gene expression data do not include a finer neuroanatomic parcellation of the cerebellum, so we could not evaluate the pattern of *TSC1* and *TSC2* expression in specific cerebellar lobules throughout development. Validating these patterns of regional gene expression in data from TSC patients and evaluating developmental patterns of *TSC1* and *TSC2* expression in different cerebellar lobules and the cortical plate will be an important goal for future work. Finally, as we have seen, because seizures are a common clinical manifestation of TSC and anti-epileptic medications are associated with cerebellar atrophy, it is challenging to separate the multiple factors that contribute to cerebellar structural changes in TSC patients.

In conclusion, we found that gene expression of *TSC1* and *TSC2* is regionally specific and developmentally dynamic, being selectively elevated in the cortical plate during fetal mid-gestation and in the cerebellum postnatally, from childhood to adulthood. We conjecture that abnormal *TSC1* and *TSC2* expression in these specific brain regions during critical developmental periods may affect key molecular pathways, result in measurable morphologic changes, and contribute to the cognitive impairment observed in a subset of the TSC population. Finally, we suggest that TSC is a promising model disorder in which to study the role of regional gene expression in brain development and disease.

## Methods

All methods are HIPAA-compliant and approved by the University of California, San Francisco Institutional Review Board (IRB). All investigations were performed in accordance with IRB guidelines.

### Allen BSI Adult Gene Expression Data

We investigated regional gene expression of *TSC1* and *TSC2* with the goal of leveraging patterns of gene expression to understand the TSC phenotype. Using a publicly available microarray dataset from the Allen Brain Sciences Institute (www.brain-map.org), we first evaluated whether *TSC1* and *TSC2* exhibit a regionally specific pattern of gene expression across the human brain. Briefly, these transcriptome data are derived from 6 adult brains and across 862 brain regions. Approximately 500 anatomically discrete samples were collected from cortex, subcortex, cerebellum, and brainstem of each of the 6 brains and profiled for genome-wide gene expression using a custom Agilent 8 × 60 K cDNA array chip. For additional details regarding the dissection methods, quality control, and normalization measures on this dataset, please see^29^and http://human.brain-map.org/. For each TSC gene, we downloaded expression values for each available probe, calculated using z-score normalization. For *TSC1*, we used probes A_24_P329635, CUST_1305_PI416573500, and CUST_1347_PI416379584. For *TSC2*, we used probes A_23_P66110, CUST_16140_PI416261804, and CUST_662_PI416408490.

### TSC1 and TSC2 expression across the developmental age spectrum

We used a spatial and temporal microarray expression data set focusing on the prenatal brain to investigate *TSC1* and *TSC2* gene expression throughout development^30^. These exon-level expression data originate from 1,340 tissue samples from both hemispheres, across 16 brain regions that span 32 consecutive periods of neurodevelopment and adulthood from 6 post-conception weeks (PCW) to 85 years (for additional details see http://hbatlas.org/). For *TSC1* and *TSC2*, we downloaded expression values, calculated using z-score normalization from the Gene Expression Omnibus (GEO) database (GSE25219).

### TSC1 and TSC2 expression in utero

We examined *TSC1* and *TSC2* expression *in utero* using data from the Allen Brain Sciences Institute. These exon-level expression data originate from four intact mid-gestational brains (two from 15–16 PCW and two from 21 PCW) and 9 layers (fetal mitotic and post-mitotic zones) as described previously (for additional details see Miller JA 2014 Nature^31^ and http://www.brainspan.org/lcm/). We downloaded expression values, calculated using z-score normalization from http://www.brainspan.org/lcm/ and evaluated the pattern of expression across the 9 fetal mitotic and post-mitotic zones, which included subpial granular layer (SG), marginal zone (MZ), outer and inner cortical plate (CP), subplate (SP), intermediate zone (IZ or inner SP), outer and inner subventricular zone (SZ), and ventricular zone (VZ).

### *In vivo* morphometric analysis of cerebellar volume in pediatric patients with TSC

With institutional review board approval, we performed a retrospective search of our institution’s clinical radiology database for MRI scans of patients diagnosed with TSC who were less than 18 years of age at the time of imaging. Informed consent was not required, as the study was retrospective in nature. We only included patients whose scans included 3D T1-weighted sequences (1 mm isotropic resolution) imaged on 3 Tesla General Electric MRI scanners. We further excluded post-operative patients and scans with excessive motion or other artifacts. We identified 28 TSC patients who met these criteria whose diagnosis was confirmed either through fulfillment of clinical diagnostic criteria for TSC or through positive genetic testing for known *TSC1* and *TSC2* mutations. We compared these TSC patients to 200 normal age- and sex-matched controls from the Pediatric Imaging Neurocognition Genetics (PING) database^32^. We performed volumetric probabilistic segmentation of the cerebellar lobules from each subject’s 3D T1-weighted scan using the SUIT (‘spatially unbiased infra-tentorial template‘) software package^33^ (see Fig. 8a for a representative example).

## Electronic supplementary material

Supplemental Information

## Data Availability

The datasets analyzed in this study are publicly available on the websites provided in the Results and Methods section of this manuscript.
