# Supplementary material for: Regionally specific TSC1 and TSC2 gene expression in tuberous sclerosis complex
Source: Sci Rep. 2018 Sep 6;8:13373. doi: 10.1038/s41598-018-31075-4 (PMC6127129; doi:10.1038/s41598-018-31075-4)
Supplement: Supplementary file 1 — Supplemental Information [file 41598_2018_31075_MOESM1_ESM.pdf]

SUPPLEMENTAL MATERIALS AND FIGURES FOR:

Regionally specific *TSC1* and *TSC2* gene expression in tuberous sclerosis complex

Yi Li, MD[1]; Matthew Barkovich, MD[1]; Celeste Karch, PhD[2]; Ryan Nillo, BA[1]; Chun-Chieh Fan, MD[3]; Iris Broce, PhD[1]; Chin Hong Tan, PhD[1]; Daniel Cuneo, BA[1]; Christopher Hess, MD, PhD[1]; William Dillon, MD[1]; Orit Glenn, MD[1]; Christine Glastonbury, MD[1]; Nicholas Olney, MD[4]; Jennifer Sachiko Yokoyama, PhD[4] ; Lucas Bonham, BS[4]; Bruce Miller, MD[4]; Aimee Kao, MD, PhD[4]; Nicholas Schmansky, MS[5]; Bruce Fischl, PhD[5,6]; Ole A. Andreassen, MD, PhD[7]; Terry Jernigan, PhD[3]; Anders Dale, PhD[3,8]; James Barkovich, MD[1,9]; Rahul Desikan, MD, PhD[1,9] and Leo Sugrue, MD, PhD[1]

[1]Neuroradiology Section, Department of Radiology and Biomedical Imaging, University of California, San Francisco, San Francisco, CA, 94143, USA
[2]Department of Psychiatry, Washington University, St. Louis, MO, 63110, USA
[3]Department of Cognitive Sciences, University of California, San Diego, La Jolla, CA, 92093, USA
[4]Memory and Aging Center, Department of Neurology, University of California, San Francisco, San Francisco, CA, 94158, USA
[5]Athinoula A. Martinos Center, Harvard Medical School, Charlestown, MA, 02129, USA
[6]Health Science and Technology Program and Computer Science and Artificial Intelligence Laboratory, Massachusetts Institute of Technology, Boston, MA, 02139, USA
[7]NORMENT Institute of Clinical Medicine, University of Oslo and Division of Mental Health and Addiction, Oslo University Hospital, Oslo, Norway
[8]Departments of Radiology and Neurosciences, University of California, San Diego, La Jolla, California, United States of America
[9]Departments of Neurology and Pediatrics, University of California, San Francisco, San Francisco, CA, 94143, USA

*Correspondence:
Drs. Yi Li and Leo Sugrue
Neuroradiology Section, L-352
University of California, San Francisco
505 Parnassus Avenue
San Francisco, CA, USA 94143
Email: yi.li@ucsf.edu, leo.sugrue@ucsf.edu
Phone: (415)-353-1079
Fax: (415)-353-8593

Genes showing strongest protein-protein and co-expression network interactions with TSC1 and TSC2

Protein-protein and co-expression network analysis showed the strongest network weights (> 0.60) between *TSC1*, *TSC2* and other components of the P13K/AKT/mTOR pathway, namely *mTOR*, *c12orf5/TIGAR*, *RPTOR*, *MLST8*, *AKT1S1*, *RHEB*, *RPS6KB1*, *DEPTOR*, *EIF4EBP1* and *RICTOR. TSC1* and *TSC2* are components of the mTOR pathway, forming an intracellular complex that suppressed mTOR through the inactivation of RHEB, an mTOR activator. RPTOR and RICTOR both complex with MTOR, and DEPTOR is an mTOR suppressor. Beyond components of the P13K/AKT/mTOR pathway, we found interactions (network weights > 0.30) between *TSC1*, *TSC2* and the cytoskeleton-related *MSN* and *EZR,* as well as the 14-3-3 cell signaling proteins *YWHAB* and *YWHAG* (Figure 7). Ezrin (EZR) is expressed in neuroblasts and mediates the genesis of neurites in developing brain and may also be important in growth of perisynaptic astrocyte processes in older animals [1]. Ezrin and moesin (MSN) are both proteins that regulate signaling pathways by binding transmembrane receptors and linking to downstream signaling components [2]. YWHAB has been shown to interact with RAF1 and CDC25 phosphatases, suggesting that it may play a role in linking mitogenic signaling and the cell cycle machinery. YWHAG (aka human 14-3-3 gamma gene) plays an important role in signal transduction leading to mitosis and cellular proliferation; knockdown leads to reduced brain size and increased diameter of the heart tube in zebrafish. Humans with mutations in YWHAG develop severe infantile seizures and hypertrophic cardiomyopathy [3].

SUPPLEMENTAL FIGURE LEGENDS:

Supplemental Figure 1. Regionally specific *TSC1* (top) and *TSC2* (bottom) expression using data from 125 disease-free adult post-mortem brains from the Genotype-Tissue Expression (GTEx) Consortium.

Supplemental Figure 2. Regionally specific *TSC1* (left) and *TSC2* (right) expression using data from 134 disease-free adult post-mortem brains from the UK Brain Expression Consortium (UKBEC).

Supplemental Figure 3. Regionally specific mTOR (a), MLST8 (b), RPS6KB1 (c), RICTOR (d) expression using data from the Genotype-Tissue Expression (GTEx) Consortium.

Supplemental Table 1. a) Linear regression model comparing volumes of cerebellar lobules in patients with TSC who are not taking antiepileptic medications (N=10) to normal controls (N=200). **b)** Linear regression model comparing volumes of cerebellar lobules in patients with TSC who are taking antiepileptic medications (N=18) to normal controls (n=200). All regression models control for subject age and gender.

SUPPLEMENTAL FIGURES

Supplemental Figure 1.

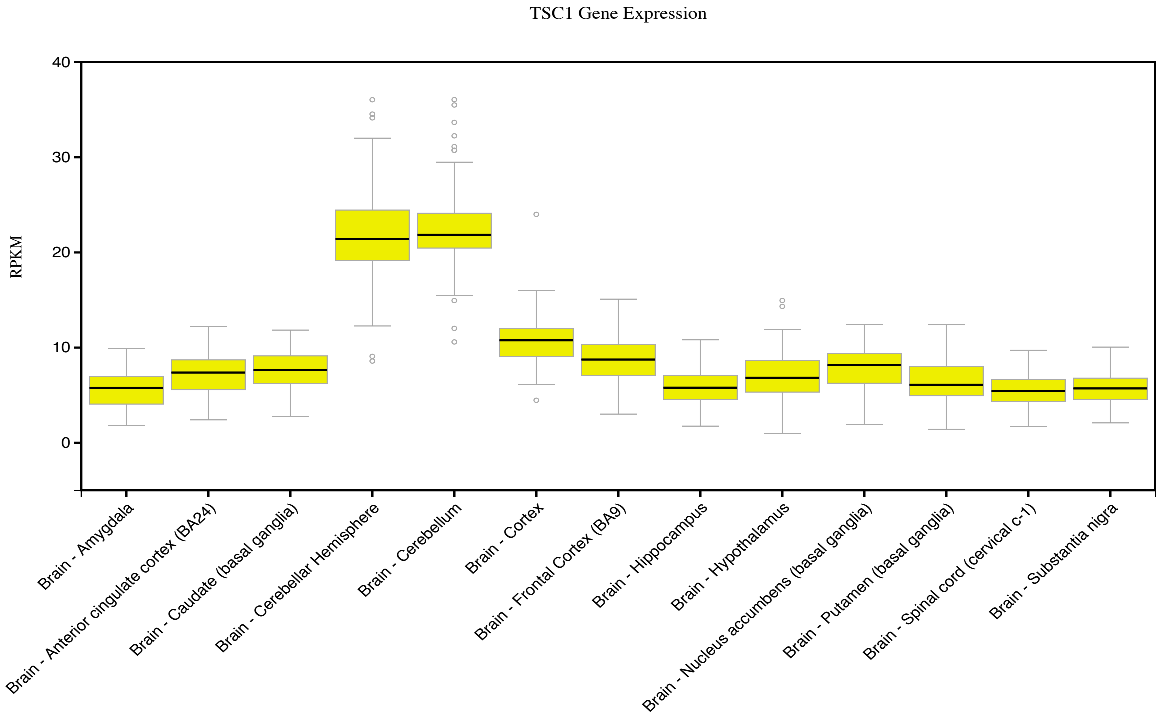

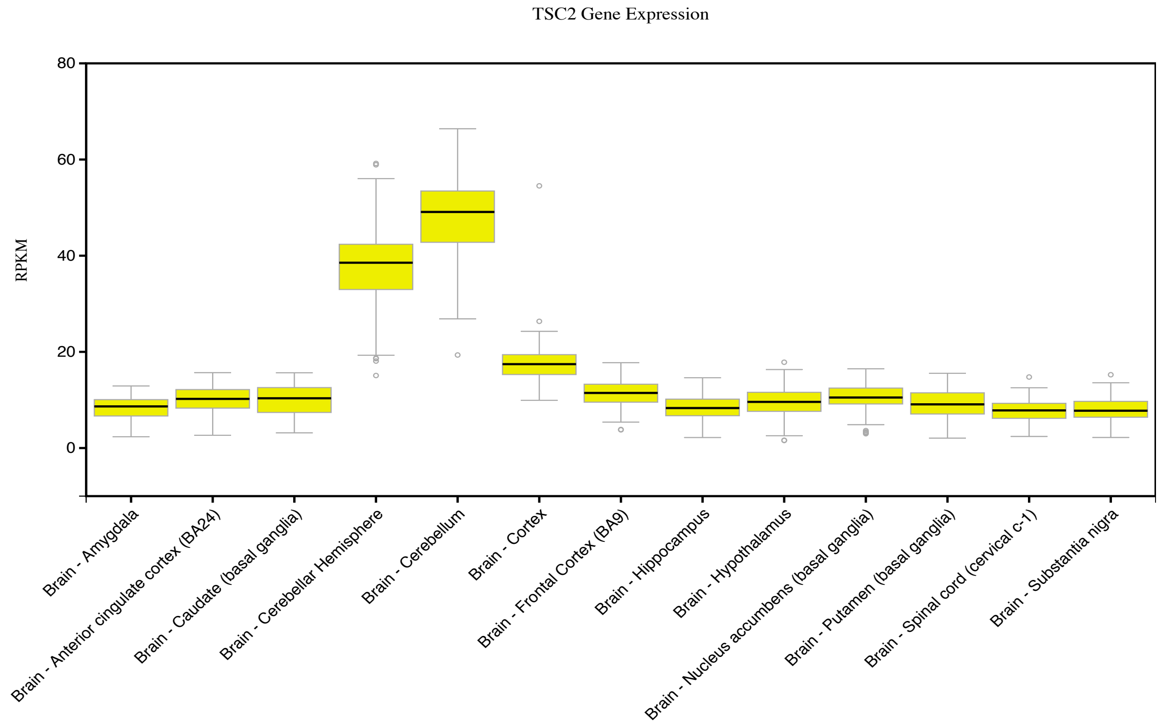

Supplemental Figure 2

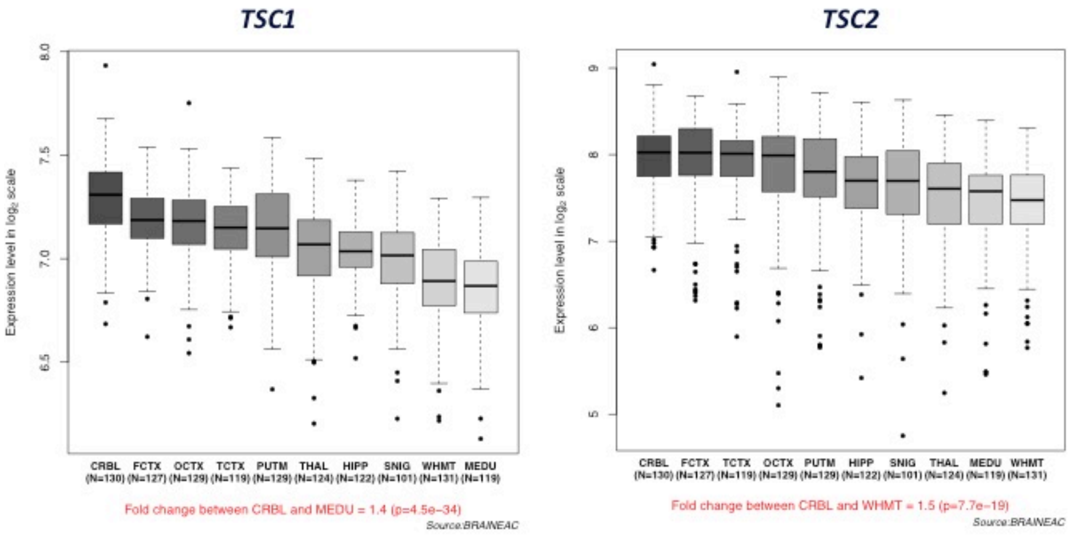

Supplemental Figure 3

a.

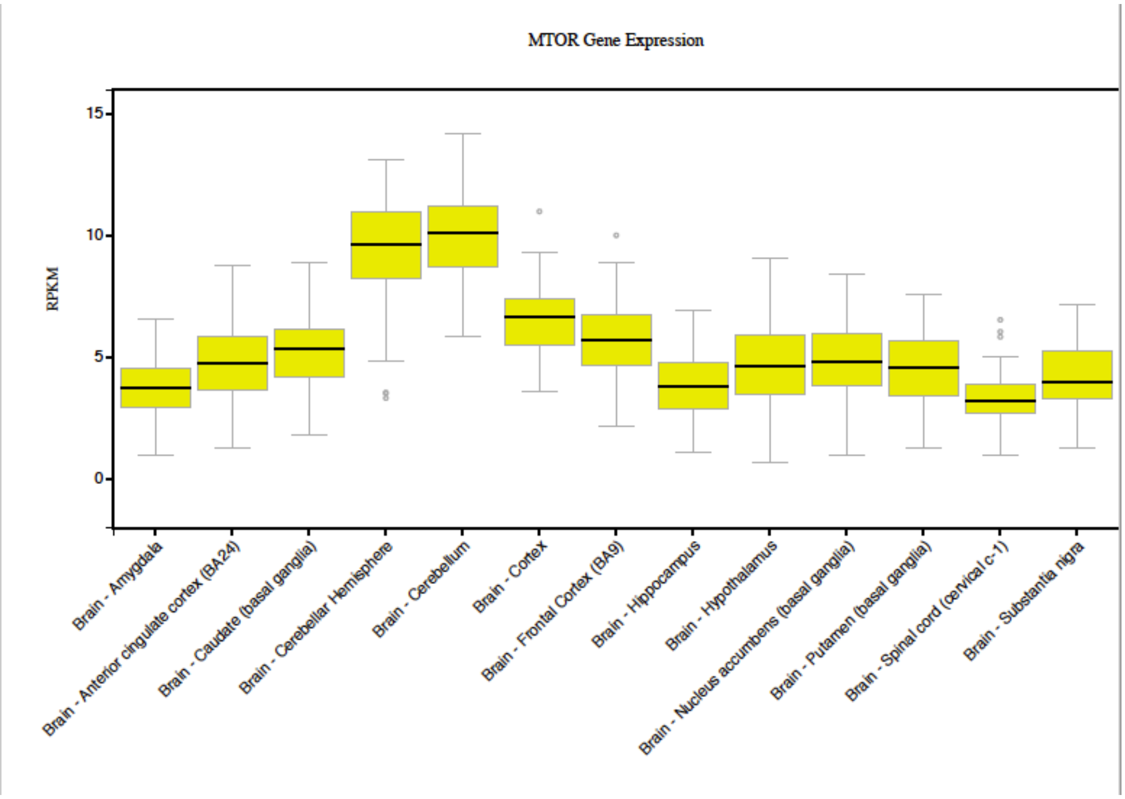

b.

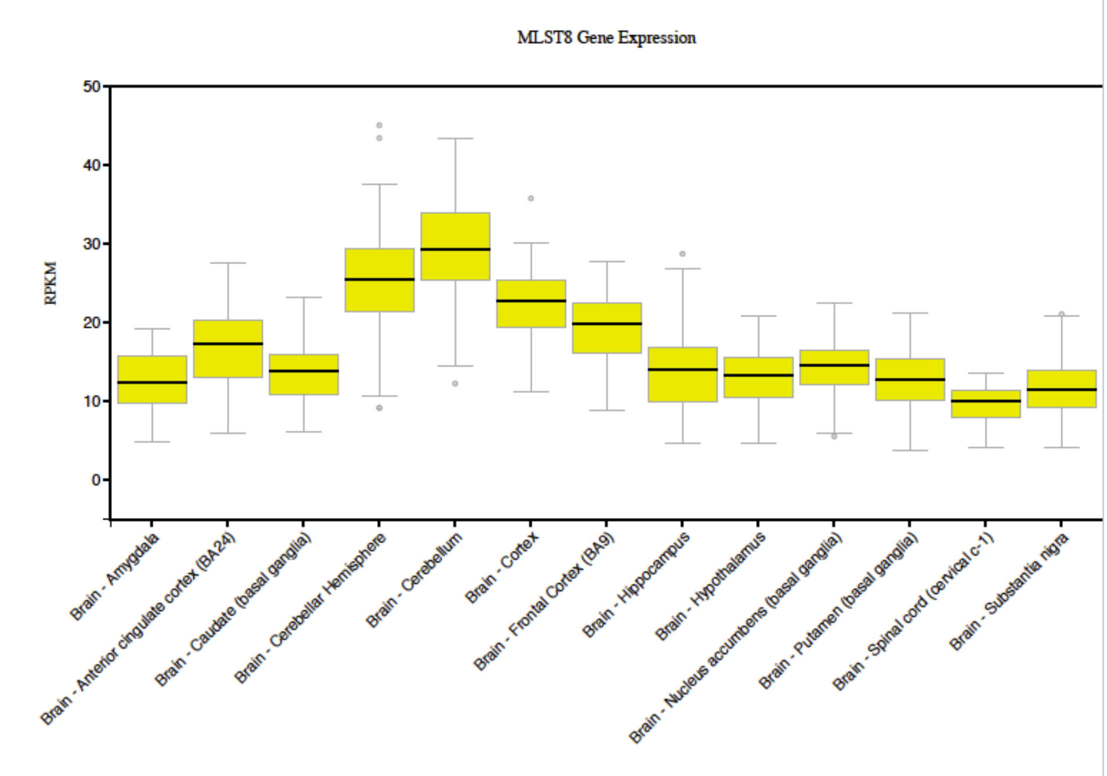

c.

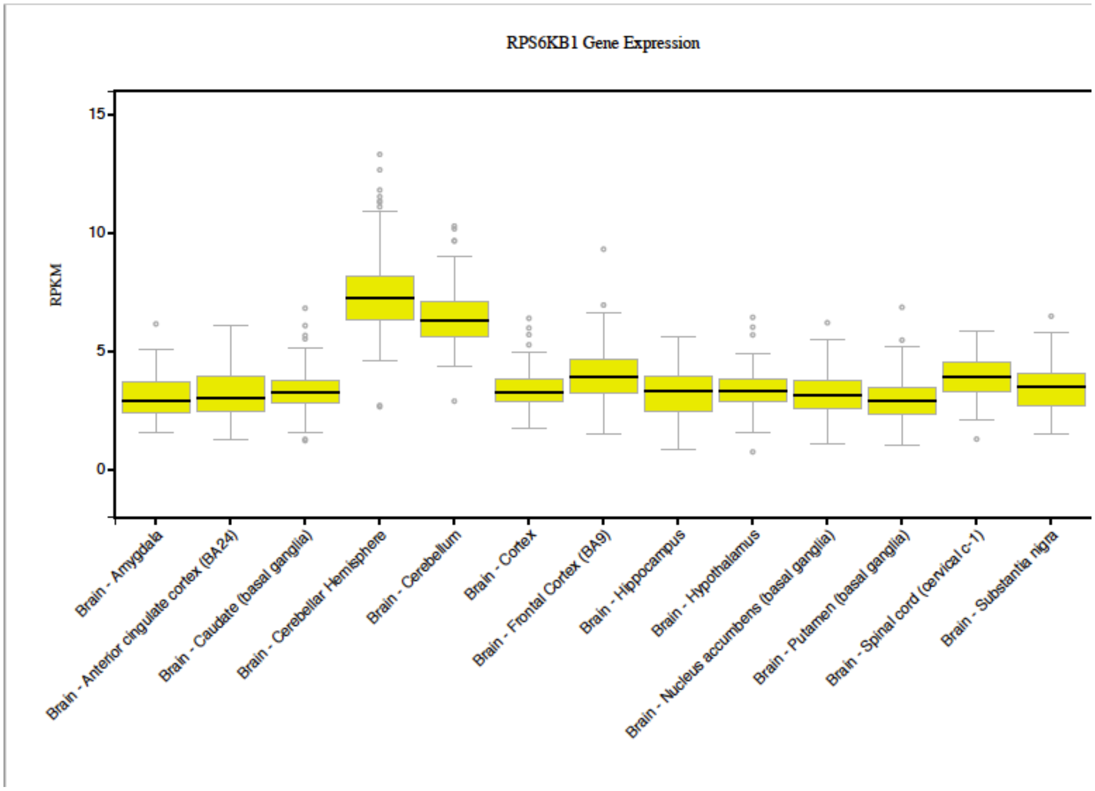

RPS6KB1 Gene Expression
RPKM
15
10
5
0
Brain - Amygdala
Brain - Anterior cingulate cortex (BA24)
Brain - Caudate (basal ganglia)
Brain - Cerebellar Hemisphere
Brain - Cerebellum
Brain - Cortex
Brain - Frontal Cortex (BA9)
Brain - Hippocampus
Brain - Hypothalamus
Brain - Nucleus accumbens (basal ganglia)
Brain - Putamen (basal ganglia)
Brain - Spinal cord (cervical c-1)
Brain - Substantia nigra

d.

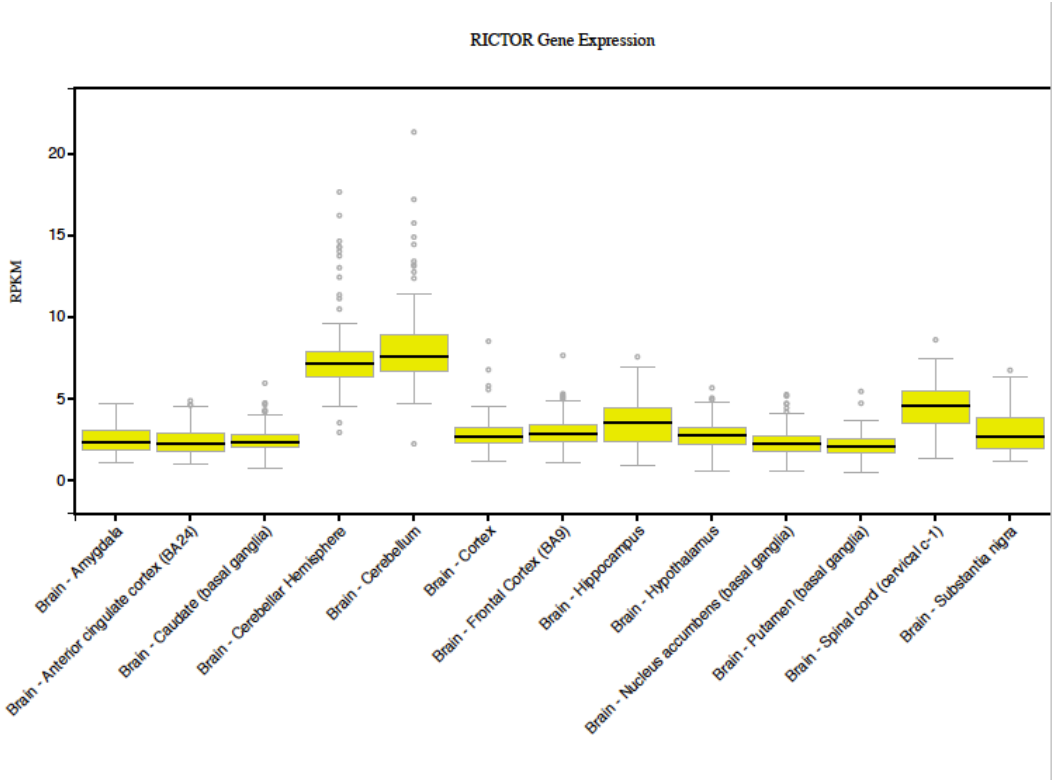

RICTOR Gene Expression
RPKM
20
15
10
5
0
Brain - Amygdala
Brain - Anterior cingulate cortex (BA24)
Brain - Caudate (basal ganglia)
Brain - Cerebellar Hemisphere
Brain - Cerebellum
Brain - Cortex
Brain - Frontal Cortex (BA9)
Brain - Hippocampus
Brain - Hypothalamus
Brain - Nucleus accumbens (basal ganglia)
Brain - Putamen (basal ganglia)
Brain - Spinal cord (cervical c-1)
Brain - Substantia nigra

Supplemental Table 1a

Cerebellar lobule	Coefficient	se	p-value
i to iv	0.02	0.01	0.02
v	0.02	0.01	0.006
vi	0.03	0.02	0.05
crus i	-0.04	0.03	0.30
crus ii	0.01	0.02	0.73
vii b	0.01	0.01	0.37
viii a	0.01	0.01	0.26
viii b	0	0.01	0.80
ix	-0.01	0.01	0.20
x	0	0	0.93

Supplemental Table 1b

Cerebellar lobule	Coefficient	se	p-value
i to iv	0.01	0	0.18
v	0.01	0.01	0.31
vi	-0.02	0.01	0.07
crus i	-0.07	0.02	0.003
crus ii	-0.05	0.02	0.002
vii b	-0.02	0.01	0.01
viii a	-0.02	0.01	0.009
viii b	-0.01	0.01	0.09
ix	-0.02	0.01	0.007
x	0	0	0.19

References

1. Moon, Y. *et al.* Expression of ezrin in subventricular zone neural stem cells and their progeny in adult and developing mice. *Histochem. Cell Biol.* **139,** 403–413 (2013).
2. Neisch, A. L. & Fehon, R. G. Ezrin, Radixin and Moesin: Key regulators of membrane-cortex interactions and signaling. *Current Opinion in Cell Biology* **23,** 377–382 (2011).
3. Komoike, Y. *et al.* Zebrafish gene knockdowns imply roles for human YWHAG in infantile spasms and cardiomegaly. *Genesis* **48,** 233–243 (2010).
